# A review of injury epidemiology in the UK and Europe: some methodological considerations in constructing rates

**DOI:** 10.1186/1471-2458-9-226

**Published:** 2009-07-10

**Authors:** Roxana Alexandrescu, Sarah J O'Brien, Fiona E Lecky

**Affiliations:** 1 Trauma Audit and Research Network, Clinical Science Building, Hope Hospital, University of Manchester, Manchester, UK; 2 School of Translational Medicine, University of Manchester, Manchester, UK

## Abstract

**Background:**

Serious injuries have been stated as a public health priority in the UK. However, there appears to be a lack of information on population-based rates of serious injury (as defined by a recognised taxonomy of injury severity) at national level from either official statistics or research papers. We aim to address this through a search and review of literature primarily focused within the UK and Europe.

**Methods:**

The review summarizes research papers on the subject of population based injury epidemiology published from 1970 to 2008. We examined critically methodological approaches in measuring injury incident rates including data sources, description of the injury pyramid, matching numerator and denominator populations as well as the relationship between injury and socioeconomic status.

**Results:**

National representative rates come from research papers using official statistics sources, often focusing on mortality data alone. Few studies present data from the perspective of an injury pyramid or using a standardized measure of injury severity, i.e. Injury Severity Score (ISS). The population movement that may result in a possible numerator – denominator mismatch has been acknowledged in five research studies and in official statistics. The epidemiological profile shows over the past decades in UK and Europe a decrease in injury death rates. No major trauma population based rates are available within well defined populations across UK over recent time periods. Both fatal and non-fatal injury rates occurred more frequently in males than females with higher rates in males up to 65 years, then in females over 65 years. Road traffic crashes and falls are predominant injury mechanisms. Whereas a straightforward inverse association between injury death rates and socio-economic status has been observed, the evidence of socioeconomic inequalities in non-fatal injuries rates has not been wholly consistent.

**Conclusion:**

New methodological approaches should be developed to deal with the study design inconsistencies and the knowledge gaps identified across this review. Trauma registries contain injury data from hospitals within larger regions and code injury by Abbreviated Injury Scale enabling information on severity; these may be reliable data sources to improve understanding of injury epidemiology.

## Background

Injury is a major, preventable public health problem in terms of morbidity, premature mortality or disability. Worldwide about 5.8 million people die every year as a result of an injury and the projections for 2020 show that 8.4 million deaths are expected annually [1,2]. Moreover, injuries are an important source of direct medical costs as well as indirect costs resulting from economic production losses; in the Netherlands for example, the direct costs of injury represents 5% of the health care budget whereas in Spain the total costs associated with Road Traffic Crashes (RTC) alone account for 1.35% of the gross national product [3,4].

In the UK, injury is the commonest cause of death in the first four decades of life and amongst the leading causes for ill-health – "for every injury death there are 45 hospital episodes, 630 doctor consultations and 5000–6000 minor injuries" [5]. In 1999 after Government intent to put all non-fatal injury (i.e. injuries defined as those requiring at least a general practitioner consultation) on the political agenda, reducing serious injury became a public health priority [6,7]. At national level official data on injury occurrence comes from health surveys, general household surveys, and morbidity surveys in general practice. For example: The Fireworks Injury Enquiries in Scotland provides information on proportions and trends in firework injuries (1975–2005) by age, place of accident or type of injury and outcome [8]; the ninth and tenth series of the Health Survey for England (2000–2001) that offer data on nonfatal accidents by socio-demographic characteristics, injury details and consequences [9,10]; the Morbidity Survey in General Practice Fourth National Study 1991–1992 [11] and the Home and Leisure Accident Surveillance Systems reports until 2002 [12] describe reasons for consultation in general practice and injury consultations in A&E departments respectively. In this context it is noteworthy the European Injury Database established in 1999, under the European Prevention Programme, as an enlargement to European Home and Leisure Accident Surveillance System. The purpose of this database is to provide access to national injury data collected from Emergency departments of Member States hospitals for the development of evidence-based injury prevention strategies [13]. English Hospital Episodes Statistics (HES) are a relatively comprehensive national statistics dataset relying on information regarding hospital discharges sent by National Health System (NHS) Trusts [14]. Using HES data Jones et al. [15] collated injury information and underlined the issues related to availability of valid denominators in constructing injury death rates and hospitalization rates; however, some approximate rates per 100,000 population by age group based on finished consultant episodes (i.e. a completed period of care a patient using a NHS hospital bed, under one consultant within one health care provider) from HES data were calculated: 1,100 (0–14 y); 1,100(15–59 y); 1,300 (60–74 y) and 4,313 (75+y). Fatal injury data in England and Wales are reported in the yearly publications of the National Office for Statistics, i.e. Mortality Statistics – Injury and Poisoning DH4 series (between 1974 to 1989 entitled Mortality Statistics – Accidents and Violence) as well as in the Decennial Supplements that deal with trends and geographic patterns [16,17]. However, in order to control injury by prevention strategies incidence rates alone are insufficient. The severity of each injury needs to be characterised as it has been shown to relate to health service resource use and economic loss, as does the causal mechanism of injury [18].

An epidemiological challenge is defining 'serious' injury using existing databases. Indicators for monitoring non-fatal serious injuries have been proposed (i.e. length of hospitalisation four or more days, or admissions for long bone fractures) and further criticised [7,19,20]. The national database (HES) uses the International Classification of Diseases ICD-10^th ^revision to categorize injuries. However, the ICD contains little indication of injury severity. On the other hand trauma registries (in the UK, Trauma Audit and Research Network (TARN) [21]), in line with international conventions, use the Abbreviated Injury Scale (AIS), which is an anatomically-based injury description system that lists over 1200 serious injuries and scores the immediate severity of each injury from one (minor) to six (maxima). Table 1 offers an example of injury description within ICD and AIS lexicons. The AIS allows computation of several measures of injury severity that have been used in trauma research. All these measures are based on the single AIS severity scores and are constructed in order to measure one's patient global severity when he/she has multiple injuries: Maximum Injury Severity Score (MAIS) which is the highest single AIS score [22,23], Injury Severity Score (ISS) calculated by summing the squares of the highest AIS-scores in three body areas [24], or the New Injury Severity Score (NISS) developed by Osler et al. in 1997 based on the three most severe lesions regardless of the body region [25]. Moreover, in the middle of the 1990s, Osler introduced the ICD-9 based ISS (ICISS) that allows severity to be classified based on the ICD-9 classification of injuries [26]. Injury Severity Score ICD-9 is a product of survival risk ratio from each injury sustained, based on the values of the survival rates of prior patients with similar diagnoses as classified by ICD-9. Hospital databases do contain ICD diagnoses and as a result there is opportunity to derive AIS severity scores ICISS for the estimation of injury severity [27]. Apart from the measures based on AIS, it should be mentioned in this context the Paediatric Trauma Score (PTS) – an injury scoring method specifically designed for children [28,29]. It relies on a six point score calculated from weight, blood pressure, ability to maintain on airway, level of consciousness and presence of fracture/wound. Several issues have been raised with regard to the use of any of these specific measures of injury severity. Injury Severity Score, MAIS and NISS for example, are 'congenitally attached' to the AIS score for calculation [30], add to this ISS does not take into account age or chronic preconditions and it is limited to one injury per body region [31,32]. Injury Severity Score, MAIS and PTS only apply to physical injury and are not suitable for other events, such as drowning. Moreover, ISS and MAIS are derived from adult norms and consequently are not 'child friendly' [33]. Although developed to correct some of the limitations related to ISS, NISS seems to be a measure 'that tends to overstate severity for lesser injuries' [31]. With regard to ICISS, there is limited evidence on the validation and performance of this scoring method with ICD10 classification of injuries [33-35]. Although there is no consensus on the 'best' method for defining injury severity, the ISS as developed by the American Association for the Advancement of Automotive Medicine remains the 'gold standard', most reliable and extensively used measure of injury severity and ' a cornerstone of injury epidemiology' [31,32,36,37]. The ISS characterises the likely threat to life from injury and is widely used in hospital trauma registries to characterise their activity and performance. However, it seems that this data is rarely related to populations to create severity-specific rates of major injury. We have reviewed the injury epidemiology literature with the purpose to present incidence rates and characteristics of injury in UK and Europe, and discuss methodology for constructing rates, including the availability of data sources.

**Table 1:** Abdominal aorta injury description using ICD9, ICD10 and AIS90.

**Lexicon**	**Injury description**	**Code^3^**
ICD-9	Abdominal Aorta Injury	902.0
ICD-10	Injury of abdominal aorta	S35.0
AIS90	Aorta, Abdominal NFS (not further specified)	520299.*4*
	intimal tear, no disruption	520202.*4*
	laceration (perforation, puncture) NFS	520204.*4*
	minor^1^	520206.*4*
	major^2^	520208.*5*

^1^superficial, incomplete transection; incomplete circumferential involvement; blood loss ≤ 20% by volume

^2 ^rupture, incomplete transection; segmental loss; complete circumferential involvement; blood loss ≥ 20% by volume

^3 ^in italics the AIS score that allows computation of injury severity score

## Methods

To conduct this review, the authors accessed the extensive bibliography of John Rylands Library, University of Manchester as well as TARN papers and reports. Medline, EMBASE, and Cochrane Library electronic databases have been examined for English language European injury papers published from 1970 to 2008. Search terms used included: 'descriptive epidemiology injury/trauma', 'injury population based study', 'injury morbidity/mortality', 'injury incidence/deaths' and 'injury surveillance'. The review presents epidemiological studies covering injuries all types of causes. Since the primary aim was to identify comprehensively the literature focusing on the epidemiology of all injury (fatal and/or non-fatal) in UK, the papers identified from the search located in England, Wales, Scotland or Northern Ireland were cross-referenced and further reviewed. Studies from Europe were included if they present the epidemiological profile of all causes of injuries. We included papers that present data in the general population as well as within specific age groups (e.g., children, adolescents). We excluded studies covering selected injury categories, for example sport related injuries, work related injuries (by location of injury), traffic accidents, assaults (by injury cause) or head injuries (by body region), as it was out of the scope of this paper to provide the epidemiological profile within a selected group of injuries. The review addresses all three levels of non-specific measures of injury severity: deaths, hospitalisations and Accident & Emergency (A&E) attendances and categorises the papers taking into account study population (e.g. children (0–14 y) and adolescents (15–19 y)/general population – all age groups) and study location (i.e. UK/other countries in Europe). Papers that identified injury data from additional resources (e.g. general practitioners offices) have also been reviewed. The results are presented in terms such as population-based rates, proportions, or temporal trends and evidence of using severity scoring methods such as ISS. The relationship between injury and socioeconomic status (SES), the subject of other published reviews focusing on socioeconomic inequalities [38] or on overall methodological issues [39], has been examined in this review only within studies that were selected as focusing primarily on injury epidemiology. Although an important and recognised areas of injury research, injury consequences such as level of disability, economic and social costs or injury -related health behaviours, are outside the scope of this paper that has been designed to determine European injury rates and levels of severity within morbidity and mortality studies.

The electronic search of databases yielded 154 papers, 122 provided by Medline, 32 from EMBASE and no relevant article from the Cochrane library. A further selection of the articles based on abstract shows that 71 were relevant to the subject of this literature review, excluding duplicates this gave a total of 48 papers.

## Results

Forty-eight articles have been reviewed: 18 focused on national level data and 30 focused on local level data; 29 papers had used mortality data, 30 papers – hospitalization data and 19 papers – A&E data. Two or more data sources have been used by 21 papers.

### UK and Ireland

Twenty-eight studies dealing with fatal and/or nonfatal injuries occurring in the UK and Ireland were identified in the literature.

Tables 2, 3 and 4 give details of epidemiological studies of injuries in children and young people identified from the search that were carried out in the UK and Ireland between 1970 and 2008. Nine studies were identified that deal with fatal injury in England and Wales [40-48]. In England, child death rates from unintentional injuries vary between 12.05/100,000 (1975–1979) to 10.33/100,000 (1980–1984) and 4.0/100,000 (2001–2003) [40,41] whereas in the overall age group 0–19 y the rates decreased from 16/100,000 (1980) to 7/100,000 (1994) [42]. Travel patterns were responsible for the decreasing in mortality rates from 1985 to 1995 [43,44]. There was no change in the incidence of intentional injury fatalities over 1980–1994 [42]. Overall the largest proportion of injury deaths occurred in boys (e.g. 69% in England and Wales 1980–1994 [42]). Blunt trauma (RTC (Road Traffic Crashes), falls, blunt assault) is the predominant injury type for severe trauma (i.e. almost 95% [45]). Overall more than half of blunt injuries deaths and/or hospitalisation are due to RTC [45-48]. Social class gradients are present in injury deaths rates (e.g. a correlation coefficient between death due to accidents and social deprivation r = 0.56 and a 17 times higher homicide rate in social class V vs. I in England, 1980–1984 [40] and 1980–1995 [42], respectively). Of note, the five basic social classes recognised by Office of Population Censuses and Surveys are described as follows: I Professional occupations; II Managerial and technical occupations; III Skilled occupations; IV Partly – skilled occupations and V Unskilled occupations [49].

**Table 2:** Epidemiological studies from the UK – childhood injuries

**Author and date**	**Type of study/data source**	**Population** **(denominator)/size**	**Level of severity**	**Epidemiological observation**	**Major findings**	**Epidemiological shortcomings**
Walsh et al., 1996 [50]	descriptive (prospective) study/Coroner's files; Hospital data	children (0–16 y)/54400 Newcastle upon Tyne, England	deaths, hospitalization and A&E	proportions, rates by severity (ISS), type, cause; correlation A&E rates and Townsend score	1990 6 deaths: 904 admissions: 11682 A&E (1660/21490 per 100,000 admission/attendance rate); some associations of hospitalisations and A&E with deprivation	1 year study and one geographical region.
Walsh & Jarvis, 1992 [55]	descriptive (retrospective) study/Office for Population Censuses Surveys; Northern Regional Health Authority	sample children (0–16 y)/153000 Northern Region England	deaths, hospitalization	proportions by type, cause; rates by severity (ISS), age, ward; correlation of rates by census ward, severity	1980–1986 Relationship of injury admission rates, deaths and severity with deprivation, e.g. relative rate of deprived vs. affluent areas 2.4 (all injuries); 3.6 (severe injuries) Highlights of the value of ISS methodology in epidemiological analysis	96.4% of the hospitals admissions identified.
Armstrong & Robson, 1992 [47]	descriptive study/Liverpool Coroner's Office data	children (0–16 y) Liverpool, England	deaths	proportions by cause, circumstances	Highlights areas of prevention: 60% pedestrian RTC (1978–1987) – half pedestrians struck by vehicles	No population based rates; data on deaths only; one region
Hippisley-Cox et al., 2002 [56]	cross sectional survey/Trent NHS regional admissions database	children (0–14 y) Trent Region, England	hospitalization	proportions by cause, age; rates by age, severity and deprivation; rate ratios for hospitals admissions	1992–1997 socioeconomic gradients (particularly children under 5 y); adjusted rate ratio (fifth highest and lowest category by Townsend scores): 3.65 pedestrian; 3.49 burns & scalds	Data on severity based on health services use; one geographical region only
Laing & Logan, 1999 [53]	descriptive study/A&E King's College, St Thomas's, Guy's hospitals registries	children (0–14 y) South-East London, England	A&E	proportions and rates by age, gender, severity; correlation of Townsend score with A&E rates	1992/1993 13820/100,000 annual attendance rate; socioeconomic gradients (even) within a disadvantaged population: a significant correlation between Townsend score and A&E attendance rate (p < 0.001)	1 year study; 4 arbitrary categories of injury severity; codification – Home &Leisure Accident Surveillance
Edwards et al., 2008 [51]	descriptive study/Hospital Episodes Statistics	children (0–15 y) England	hospitalization	rates and proportions by cause, rate ratio by socio-economic classes, location, index of multiple deprivation, regression (injury rates- census variables)	1999–2004 1340/100,000 all injury rates 15.8/100,000 serious injury rate; falls account for 36% and 41% of all/serious admissions; socio-economic gradients for serious injuries, e.g. RR = 4.1 pedestrian i and RR = 3.0 cyclists most vs. least deprived areas	Serious injury defined by six ICD groups (S72.0, S06.1–.9, S14, S22.4, T71, T68)
Avery et al., 1990 [40]	descriptive study/Office for Population Censuses Surveys	children (0–14 y) England and Wales	deaths	rates by geographical areas, trends and correlation of deprivation with deaths rates	1975/1979 12.05/100,000; 1980/1984 10.33/100,000 geographical variations (higher urban vs. rural areas, NW vs. SE England); 1980–1984 socioeconomic gradients r = 0.56 for accidental deaths rate	Data on deaths only

**Table 3:** Epidemiological studies from the UK – childhood injuries

**Author and date**	**Type of study/data source**	**Population** **(denominator)/size**	**Level of severity**	**Epidemiological observation**	**Major findings**	**Epidemiological shortcomings**
Roberts et al., 1996 [46]	descriptive study/Office for Population Censuses Surveys	children (0–15 y) England and Wales	deaths	rates by social class and cause; trends of mortality rates	1979–83 to 1989–92 Social class gradients in mortality: 21% and 2% decline in social classes IV and V (47.5 to 37.8/100,000 and 84.7 to 82.9/100,000); 32% and 37% decline in social classes I and II (24.2 to 16.5/100,000 and 25.0 to 15.8/100,000)	Data on death only; missing data for 1981
DiGuiseppi et al., 1997 [43]	descriptive study/Office for National Statistics	children (0–14 y) England and Wales	deaths	proportions and trends of rates per mile travelled by age, gender, type road user	Travel patterns responsible for (34%) decline in children rates 1985–1992; declines in walking/cycling activities (37% and 38% declines pedestrian/cyclist rates)	Data on deaths only, focused on road traffic injuries; no population based rates for all injuries
Edwards et al., 2006 [41]	descriptive study/Office for National Statistics	children (0–15 y) England and Wales	deaths	rates and proportions by socio-economic classes, year, 3 y average, cause	1979–2003 Decline in death rates (per 100,000) from 11.1 (1979,1980, 1982) to 4.0 (2001–2003). Socio-economic gradients e.g. 13.1 times higher all external causes injury rates NSSEC* class 8 vs.1	Data on deaths only; lack of 1980 injury deaths data
Lyons et al., 1995 [57]	descriptive study/West Glamorgan injury database	children (0–14 y)/370000 West Glamorgan County, Wales	A&E	proportions by place; rates and correlation of distance to A&E, no car and Townsend with rate ratio	1993 18200 injuries/100,000 Association of overall and home injury with proximity to A&E unit; no association of injury with socioeconomic status	Fractures as a proxy indicator for severe injuries – Nuffield Hospital Classification 1 year study
Graham et al., 2004 [52]	descriptive (prospective) study/Crosshouse Hospital questionnaire data; Procurator Fiscal	children (0–13 y)/10697 Kilmarnock, Scotland	deaths, hospitalization and A&E	proportions by type; rates of admissions by age	1999/2000 5.6 hospital admissions per day Information on local injury data and preventive measures in use (cycle helmets used in 26% of cycle incidents; adult supervision in 49% of incidents)	No population based rates, no information on severity; 12.9% response rate
MacInnes & Stone 2008 [54]	descriptive study/Royal Hospital Sick Children database	children (<7 y) Glasgow, Scotland	A&E	proportions by age, gender, location, circumstances, cause, type of injury; rates by age, gender, location	1997–2001 14400/100,000 per year A&E attendance rate, peak values within 12–35 months; leading causes and types: 41% falls; 68% home location; 62% play related; 52% head injuries.	No information on severity; one geographical region only
Ness et al., 2002 [58]	descriptive survey/Glasgow Royal Infirmary Questionnaire data	children (13 y)/1493 Glasgow, Scotland	A&E	proportions by age, postcode – deprivation (Carstair Depcat)	1990 injuries by type, location (facial laceration, radius/ulna fractures most frequently; 72% outside house); most of injured children come from highest area of deprivation	53%questionnaire response rate; selection 10% of the questionnaires for analysis; study period – 3 months

* National Statistics Socio-Economic Classification (NSSEC) is a new, occupational based classification that replaced starting with 2001 the social classes. The analytic eight class version is described as follows: 1 higher managerial and professional occupations, 2 lower managerial and professional occupations, 3 intermediate occupations, 4 small employers and own account workers, 5 lower supervisory and technical occupations, 6 semi-routine occupations, 7 routine occupations, 8 never worked, long term unemployed [41]

**Table 4:** Epidemiological studies from the UK – injuries in young people

**Author and date**	**Type of study/data source**	**Population** **(denominator)/size**	**Level of severity**	**Epidemiological observation**	**Major findings**	**Epidemiological shortcomings**
Roberts et al., 1998 [42]	analytical study/Office for National Statistics	children and teenagers (0–19y) England and Wales	deaths	proportions by age, gender, type, cause, trends; rates by socio-economic classes, RR (manual/non-manual), correlation homicide and deaths of undetermined intent	1980–1995 declining trends for unintentional injury (16 to 7/100,000) and no declining trends for intentional injury (2/100,000); socioeconomic gradients (e.g. homicide rate social class V is 17 times higher than for children social class I)	Data on deaths only
Roberts et al., 1998 [48]	descriptive study/Office for National Statistics; NHS data	children and teenagers (0–19y) England and Wales	deaths, hospitalization	proportions, rates by age and cause, trends of mortality rates and costs	1992 8.6 deaths/100,000 (unintentional injuries); 1.2 deaths/100,000 (intentional) Socioeconomic gradients, declining trends for unintentional injury 1980–1995	Few data on morbidity – no population based rates
DiGuiseppi et al., 1998 [44]	descriptive study/Office for National Statistics	teenagers (15–19y) England and Wales	deaths	proportions and trends of rates per mile travelled by gender, type road user	Travel patterns responsible for (32%) decline in teenage rates 1985–1995; declines in motorcycling (12.1 to 2.5 boys; 1.4 to 0.0 girls), walking (3.2 to 2.1 boys; 1.8 to 0.6 girls) and cycling activities (1.7 to 1.1 boys; 0.4 to 0.1 girls) rates per 100,000	Data on deaths only, focused on road traffic injuries; no population based rates for all injuries
Roberts et al., 1996 [45]	analytic study Major Trauma Outcome Study	patients (0–24y)/3320 England, Wales, Northern Ireland	deaths, hospitalization	proportions by cause, type, severity (ISS ≥ 16), case fatality, trends in odds of death	1989–1995 16% decline per year of odds of deaths; case fatality (ISS ≥ 16) 50 to 8.8% 0–4y; 29.5 to 16.2% 5–14y; 29.7 to 20.4% 15–24y.; role of hospital care in the reduction of trauma mortality in young people	Data on blunt trauma only; no population based rates; analysis focused on mortality (ISS ≥ 16); pre-hospital deaths not available

Three articles deal with hospitalisation data alone or with data on death and hospitalisation, four articles focus on A&E attendances and two articles present injuries from all three levels on severity [50-58]. Injury hospitalisation rates per annum are available from two papers, i.e. 1660/100,000 in Newcastle upon Tyne, 1990 [50] and 1340/100,000 in England 1999–2004 [51]. The number of hospital admissions per day, in a Scottish district hospital, was 5.6, 1999/2000 [52]. Accident and Emergency rates vary from 13820/100,000 (1992/1993) South East London [53] to 14400/100,000 (1997/2001) Glasgow [54] and 21490/100,000 (1990) Newcastle upon Tyne [50]. Both hospital admissions and A&E attendances are higher in children 1–4 y [53,55]. Accident and Emergency attendance rates are higher in boys (e.g. boys/girls Rate Ratio 1.6 [53]). Falls are a major cause for both hospitalisations (e.g. 36.1% 0–4 y, 44.9% 5–14 y [56]) and A&E attendances (e.g. 42.3% 0–4 y, 47.7% 5–14 y [53]). Injury rates by severity (ISS) are only described in two articles and show major differences by age, place of residency, cause and type, e.g. areas (electoral wards) with highest all injury rates are not always those with the highest severity or injury death rates, over 70% of fractures are classified as 'severe' injuries [50,55]. There are inconsistencies with regard to the relationship between incidence rates and SES (for example, a significant relationship (p < 0.001), 'socioeconomic distribution' vs. no association (r = 0.06) of injury A&E attendance with SES [53,57,58]).

Tables 5 and 6 give details of injury studies carried out in well-defined communities (not restricted to children and adolescent but dealing with the population as a whole) based on geographical areas in UK and Ireland. Both fatal and non-fatal injuries have been subject of six papers, two papers present data on hospitalisation only whereas the other two articles focus on A&E attendances [59-68]. Over 1988–1991 the annual crude injury death rate was 35/100,000 for the general population Kent, East Sussex and South-Eastern London [59]; the hospitalisation rates vary from 1057.5/100,000 for the previous regions [59] to 1061/100,000 for the Welsh population, 1997–1999 [60]. A&E rates vary from 19620/100,000 for the population resident EH 45 postcode (Livingstone, 1995–1996) to 22000/100,000 within one rural area (Northern Ireland, 1986) [61,62]. An annual (1990/1991) crude incidence rate of 23.2/100,000 based on hospitalisation for major trauma (ISS>15) in Northern Ireland is available from one article [63] whereas the same figure for blunt injuries only in Mersey Region and North Wales over 1989/1990 was found to be of 19/100,000 [64]. Studies based on trauma registry data show a reduction in severity adjusted odds of deaths over 1989 to 2000 [65,66]. Road traffic crashes followed by falls are the main causes for injury deaths the reverse is true for the hospital admissions (e.g. RTC 42.1% and falls 17.2% (deaths) respective falls 42.9% and RTC 14.5% (hospitalisation), Ireland, 1980–1996/1993–1997 [67]; RTC 41.9% and falls 18% (deaths), Ireland, 1980–2000 [68]). Limb fractures (27%), poisonings (14%) and intracranial injuries (11%) were most coded admissions [59], whereas fractures/dislocations (25–30%) and sprains/soft tissue injuries (20–25%) were the leading causes for A&E attendances [61]. The rates of injury hospitalisation and A&E attendance in males exceeded those in females up to 65 y, after this age the pattern reverses [59,61]. No consistent results have been shown with regard to the relation between injury A&E attendances and deprivation (no association with deprivation (i.e. variables 'no car', 'overcrowded', 'social class 5', 'under 5 yr olds' [62] vs. association with deprivation (by Carstairs Deprivation Category (Depcat)) 20910/16630 per 100,000 for most deprived/affluent Depcat [61]).

**Table 5:** Epidemiological studies from the UK and Ireland- injuries in the general population

**Author and date**	**Type of study/data source**	**Population** **(denominator)/size**	**Level of severity**	**Epidemiological observation**	**Major findings**	**Epidemiological shortcomings**
Gorman et al., 1999 [61]	descriptive study/St John's hospital A&E data	general population/44224 (residents EH45) Livingston, England	A&E	proportions by age, gender, type and location of injury; rates by age, gender, deprivation (Carstairs Depcat), eye injury data by location and type	1995–1996 19620/100,000 attendance rate Evidence of injury association with deprivation: 20910/100,000 vs. 16630/100,000 most deprived vs. most affluent Depcat and travel distance: 21480/100,000 i.e. highest attendance rate in the Depcat 4 were the hospital is located	No information on severity of injury; one geographical region
Cryer et al., 1996 [59]	descriptive study/Office for Population Censuses Surveys; South East Thames Regional Health Authority hospital data	general population/3.67 million Kent, East Sussex, South East London, England	deaths, hospitalization	proportions of deaths by injury location; rates by age, gender, cause, ICD code	A comprehensive picture (1988–1991) on the epidemiology of injury, priority setting 35/100,000 crude death rate/1057.5/100,000 hospitalisation rate; admissions by nature of injury: fracture limb 27%, poisoning 14%, intracranial injury 11%	No information on severity; one geographical region
Gorman et al., 1995 [64]	descriptive study/Coroner's data; Home Office data; A&E, ITU, theatre registers	general population/3.2 million Mersey Region and North Wales, UK	deaths, hospitalization and A&E	proportions and rates by age, cause, ISS, injury parameters (e.g., GCS, systolic blood pressure), hospital, outcome (died/alive)	A Level I Trauma Centre (American-style) might be not sustained by blunt injury incidence (ISS>15) in region i.e. 19/100,000 for patients arriving alive at hospital 1989/1990	Only injuries ISS >15
Lecky et al., 2000 [65]	descriptive study/TARN	patients/91602 England, Wales, Northern Ireland	deaths, hospitalization	proportions by cause, process of care (prehospital timing), trends in odds of deaths, Ws*, regression (odds of deaths – Revised Trauma Score, ISS)	6% statistical significant gradual decline in case mix adjusted odds of deaths 1989–1997 RTC 36.3%, falls 46.5%	Trauma registry not whole population used as denominator; non-thermal blunt trauma; pre-hospital deaths not available
Lecky et al., 2002 [66]	descriptive study/TARN	patients/129979 England, Wales, Northern Ireland	deaths, hospitalization	proportions by age, gender, ISS, process of care (seniority of doctors), trends in odds of deaths, Ws*, regression (odds of deaths – Revised Trauma Score, ISS)	No significant change in case mix adjusted odds of death 1994–2000 (p = 0.35) 6.2% death outcome	Trauma registry not whole population as denominator; non-thermal blunt trauma pre-hospital deaths not available

*Ws provides a measure of excess survivors or deaths per 100 patients treated at each site (hospital)

**Table 6:** Epidemiological studies from the UK and Ireland- injuries in the general population

**Author and date**	**Type of study/data source**	**Population** **(denominator)/size**	**Level of** **severity**	**Epidemiological observation**	**Major findings**	**Epidemiological shortcomings**
Lyons et al., 2003 [60]	descriptive study/Patient Episode Database for Wales	general population/2.84 million Wales	hospitalization	proportions by age, type, cause; crude and world standardised rates by age, cause, deprivation category (Townsend score), hospitalisation ratios	1997–1999 1493/100,000 (world) standard admission rate; socioeconomic gradients in children and older people for pedestrian assault related injuries	No information on severity of injury
McKee et al., 1990 [62]	descriptive study/Hospital A&E data	sample of general population – one rural area Northern Ireland	A&E	rates; regression (A&E attendance – distance travelled, socio-economic variables)	1986 22000/100,000 attendance rate association of injury with travel distance to A&E (r = -0.73); no association with deprivation or practice characteristics	Extrapolation of results less likely, one geographical region
McNicholl & Cooke, 1995 [63]	descriptive study/Northern Ireland hospitals records	general population/1 million Northern Ireland	hospitalization	proportions and rates by age, gender, cause, diagnosis, process of care (surgical procedures), outcome (death, persistent vegetative state, severe/moderate disability, good recovery)	1990/1991 23.2/100,000 incidence rate overall/20.5/100,000 excluding terrorist activities (injuries ISS >15)	1 year only study, only injuries ISS>15 (excludes pre-hospital deaths)
Boland et al., 2005 [68]	descriptive study/Central Office Statistics; Hospital In Patient Enquire data	general population Republic of Ireland	deaths, hospitalization	proportions, standardised mortality and admission ratios by cause, age, gender, urban vs. rural	1980–2000/1993–2000 unintentional injuries Standardized mortality/admission ratios (rural) 103.0/104.6	No information on severity
Scallan et al., 2004 [67]	descriptive study/Central Statistics Office; Hospital In Patient Enquire	general population Ireland	deaths, hospitalization	proportions and rates by cause, type	Highlights the importance of using morbidity (1993–1997, 1239.9/100,000) & mortality (1980–1996, 31.6/100,000) data on a complementary way; 1:39 deaths: hospital admissions ratio	Morbidity and mortality data cover different time periods; unintentional injuries

### Europe

Twenty studies [69-88] dealing with fatal and/or nonfatal injuries occurring in different European cities and countries were identified in the literature (tables 7, 8, 9 and 10). Nine focused on children and adolescents [69-77].

**Table 7:** Epidemiological studies from Europe – childhood and teen injuries

**Author and date**	**Type of study/** **data source**	**Population** **(denominator)/size**	**Level of severity**	**Epidemiological observation**	**Major findings**	**Epidemiological shortcomings**
Brudvik., 2000 [76]	descriptive study/Haukeland University Discharge Register	children<16y/227250 Bergen Norway	hospitalization and A&E	proportions by gender, body region, cause, circumstances of injury; rates by age, cause	1998 Annual injury incidence 9% preschool children/13% school children head injury (51%) in preschool children upper extremity injury (46%) in school children 63% of injuries occurs outdoors	No information on severity; 1 year study and one geographical region only
Mattila et al., 2005 [69]	descriptive study/Finnish Official Cause of Death Statistics	adolescents (10–19y)/0.8 million (1971) - 0.6 million (2002) Finland	deaths	proportions and rates by age, gender, year, trends of mortality rates	1971–2002 decline in death rates from 43.0 to 19.9 per 100,000; decline in RTC; no changes in intentional deaths	Data on deaths only
Parkkari et al., 2000 [71]	descriptive study/Finnish Official Cause of Death Statistics; National Hospital Discharge Register	children (0–14y)/1.1 million (1971) - 0.9 million (1995) Finland	deaths, hospitalization	rates and proportions by age, gender, cause type, year; trends of incidence and mortality rates	1971–1995 decline of incidence fatal injury rates (per 100,000): 20.1 to 4.6 (girls)/36.7 to 9.3 (boys); 1995 causes of deaths: 41% RTC, 24% intentional injuries, 12% drowning; little change non-fatal injuries rates	No information on severity; serious injuries defined by those requiring hospitalisation
Stefansdottir & Mogensen, 1997 [70]	descriptive (retrospective) study/Reykjavik City Hospital data	children (0–14y)/20756 Reykjavik, Iceland	deaths, hospitalization and A&E	proportions by age; rates by age, gender and trends of incidence rates	1974–1991 Total incidence rate 760 per 100,000/year 1987–1991 Mortality rate 6.5 per 100,000/year	No information on severity; one geographical region only
van der Voorde et al., 2008 [72]	descriptive study/PaEdiatric Network around Trauma	children (0–17y)/1.2 million Flanders, Belgium	deaths, hospitalization and A&E	proportions by age, gender, cause, severity (ISS), type, body region, location and injury circumstances, process of care (e.g., waiting time); rates by cause	2005 11900/100,000 all A&E injury rate; 1150/100,000 A&E traffic accidents rate 84.3% blunt injuries, 40.6% home injuries, 53.8% sport injuries 37.3% of all injuries have ISS>8	No population based rates of major trauma (ISS>15), 'severe trauma' defined by length of hospital stay>48 hours, including all non-survivors, 1 year study, and one geographical region only; analysis based on 21.9% sample

**Table 8:** Epidemiological studies from Europe – childhood and teen injuries

**Author and date**	**Type of study/data source**	**Population** **(denominator)/size**	**Level of severity**	**Epidemiological observation**	**Major findings**	**Epidemiological shortcomings**
Borzecky et al., 2002 [77]	descriptive study/Surgical Ward of Specialist Paediatric Hospital Kielce data	children (7–15 y) Kielce, Poland	hospitalization	proportions by age, gender, cause, location, urban/rural, time of occurrence	1997/1998 46% of injuries within age 10–12 y; most of injuries occurs in boys; leading locations: 56% at home; 18% on the road; 13% at school, 7% at farms; 53% rural area	No population based rates, no information on severity; 1 year study and one geographical region only
Oprescu et al., 2008 [73]	analytic study/Children Clinical Hospital data	children (0–18 y)/Cluj Napoca, Romania	A&E	proportions by age, gender, type, cause, ethnic status, location, process of care (e.g. waiting time), OR (injury type/age, gender, location); rates by age	1999–2003 A&E attendance rate per 100,000: 197 (age <5 y)/140 (age 5–14 y)/135 (age 15–18 y) 77.8% unintentional injuries,; 55.8% home injuries (unintentional); higher risk of injuries for boys vs. girls; falls as a leading cause age 0–14 y	No information on severity; one geographical region only; non-fatal injuries
Petridou et al., 2001 [75]	analytic study/Emergency Department Injury Surveillance System	children (0–14 y) Athens, Magnesia, Island of Corfu Greece	A&E	proportions by age, gender, type, circumstances of injury, process of care (e.g. treatment), rates and regression (OR intentional vs. non-intentional -socio-demographic variables, injury details)	1996–1997 108 injuries out of 46.807 due to violence; rates per 100,000 (violence): 18 (Athens)/21 (Magnesia)/31 (Corfu) subgroup at higher risk: boys/age 10–14 y/migrants, OR = 1.3/2.7/3.6	No population based rates for un-intentional injuries
Petridou et al., 2005 [74]	analytic (prospective) study/Health care outlets and educational institutions; police records; hospital data	children (0–14 y)/784 Velestino town, Greece	hospitalization, A&E and other sources – police, health care outlets)	proportions, rates by severity (ISS), age, gender; OR, regression (OR injury – socio-demographic and somatometric variables)	1994/1995 28.2 per 100 person year overall incidence rate; 6.3 per 100 person year incidence rate (ISS>4) subgroup at higher risk: children with younger parents/low level of education, OR = 1.33/1.37	1 year study and one geographical region only

**Table 9:** Epidemiological studies from Europe – injuries in the general population

**Author and date**	**Type of study/** **data source**	**Population** **(denominator)/size**	**Level of** **severity**	**Epidemiological** **observation**	**Major** **findings**	**Epidemiological** **shortcomings**
van der Sluis et al., 1996 [85]	descriptive study/University Hospital Groningen data	youth (20–29 y) and elderly (>60 y) Groningen, The Netherlands	hospitalization	proportions by severity (ISS), age, gender, cause, body region, process of care (e.g. length of stay), outcome (disability, died, vegetative state)	1985–1990 (injuries ISS ≥ 16) RTC leading cause 76.6 vs. 79.3% young vs. elderly; 19.6 vs. 38.8% mortality in young vs. elderly patients 100% mortality in elderly with ISS ≥ 50	No population based rates; only injuries ISS ≥ 16
van Beeck et al., 1998 [84]	descriptive study/Road Traffic Accident Registry; Occupational Registry; National Medical Registry; Dutch Central Bureau of Statistics	general population The Netherlands	deaths, hospitalization and A&E	proportions and rates by cause, location, trends of crude/standardized mortality, incidence and case-fatality rates, exposure/injury risk	1950–1995 (several data sources) Mortality upward trend (1950–1970), downward trend until mid 80s then diminishes; all changes reflecting trends in incidence and case-fatality rates. Role of trauma care, preventive measures, economic & autonomous factors (cultural, demographical and technological trends)	Injury severity defined by case fatality within broad classes, e.g. intracranial injuries; internal injuries organs in the chest
Kannus et al., 2001 [88]	descriptive study/National Hospital Discharge Register	adults (>15 y)/5 million Finland	hospitalization	proportions and rates crude/standardized by gender, cause, mechanism, trends of incidence rates	Changes in the profile of injury (1971–1995) with falls as the leading cause for both men and women 1995 falls age adjusted incidence rate male/female 635/689 per 100,000	No information on severity; serious injuries defined by those requiring hospitalisation
Kannus et al., 2005 [87]	descriptive study/Finnish Official Cause of Death Statistics	adults (>15 y)/3.5 million (1971) 4.3 million (2003) Finland	deaths	rates crude/standardized by age, gender, cause, trends of mortality rates	Changes in the unintentional injury deaths (1971–2003) with falls replacing RTC as the leading cause 1971–2003 falls age adjusted death rate male/female 18–24/30-18 per 100,000	Data on deaths only; unintentional injuries
Sahlin et al., 1990 [81]	descriptive study/Trondheim hospital records and questionnaire data; general practice data	general population Trondheim, Norway	hospitalization general practitioners visits	proportions by severity (AIS code, 1976), body region, location; rates by age, gender, type	1985/1986 incidence rate 11400 per 100,000 persons; 0.4% of all injury – fatal; 9% of all injury – hospitalised Home accidents leading cause for injuries (39%) followed by RTC (15%) RTC leading cause for fatal accidents (42%)	1 year study and one geographical region only

**Table 10:** Epidemiological studies from Europe – injuries in the general population

**Author and date**	**Type of study/data source**	**Population** **(denominator)/size**	**Level of severity**	**Epidemiological observation**	**Major findings**	**Epidemiological shortcomings**
Ekman et al., 2007 [79]	descriptive study/National Statistical Offices; WHO database	general population/98150 Sweden (Boras); 65841 Latvia (Jelgava); 378913 Lithuania (Kaunas); 101140 Estonia (Tartu) Sweden, Baltic States	deaths	rates by gender, crude and standardized, yearly, 3 y average; trends of mortality rates	1990–2002 mortality rates per 100,000: 38 (Boras)/101 (Tartu)/112 (Jelgava)/126 (Kaunas); stable trends in Sweden, increasing rates until 1994, seamed to stabilized after 1997 in Baltic communities; higher rates in males vs. females and in age group under 65 y old in the Baltic communities than in Boras, Sweden.	Data on death only
Buschmann et al., 2008 [86]	descriptive study/German trauma registry	general population (0–55 y) Germany	deaths, hospitalization and A&E	proportions by age, gender, body region, cause, process of care (e.g., length of stay) outcome (died/alive)	1997–2003 children 3% of all patients multiple injured 0–15 y: 61% boys vs. 39% girls; over 70% head injuries 0–55 y: 41.3% RTC, 59.5% thorax injuries	No population based rates, only injuries ISS>16; data focused on children
Tiret et al., 1989 [83]	descriptive study/Hospitals data; deaths certificates	general population/2.7 million Aquitaine, France	deaths, hospitalization	proportions by severity (ISS), cause, type, outcome (eight days still hospitalised/died in hospital); rates by age, gender, cause; non-fatal/fatal rate ratio	1985/1986 136/10,000 all injury incidence rate; 40% falls, 27% traffic accidents, 15% poisonings Origin: suicide 14%, assault or homicide 3%, 82% others	1 year study and one administrative region only
Di Bartolomeo et al., 2004 [78]	descriptive (prospective)study/Friuli Venezia Giulia regional registry	general population/1.2 million Friuli Venezia Giulia Italy	deaths, hospitalization	proportions by age, gender, cause, severity (ISS), process of care (e.g., timing), outcome (died/alive), rates by severity	1998/1999 238 per mil per year mortality rate; 522 per mil per year incidence rate for severe injuries (ISS>15 & pre-hospital deaths); 98.2% blunt injury 81% RTC, 9.1% falls	Only injuries ISS >15; 1 year study and one geographical region only (excludes self inflicted injuries)
Plasencia & Borell 1996 [82]	cross sectional survey/A&E Hospitals questionnaire data; City Death Registry	adults (>14 y)/1.7 million Barcelona; 6 million Catalonia, Spain	deaths, hospitalization and A&E	proportions by age, gender, cause, severity (ISS), type, location; rates age, gender, cause, location; case admission ratio	1990/1991 7470/100,000 all injury rate 1.4 times higher rates in males vs. females, falls as a leading cause; 56/100,000 mortality rate 2 times higher in males vs. females; traffic injuries as leading cause; 4% of all injuries have ISS>8; 1 death:6 admissions:133 A&E	No population based rates of major trauma (ISS>15, 1 year study, and one geographical region only; analysis based on extrapolated data on injuries
Petridou et al., 2004 [80]	descriptive study/Questionnaire data; A&E Injury Surveillance System	adults (>15 y) Greece	hospitalisation, A&E	proportions by age, gender, type; rates by gender, event timing (injury in relation to the interview date)	2001 5.9 per 100 person-year incidence reported within a survey vs. 12.9 per 100 person-year incidence reported from the surveillance system	Major injuries defined as those requiring health care; 1 y study

Child injury mortality rates vary from 43.0 to 19.9/100,000 over 1971–2002 in Finland [69] to an overall 6.5/100,000 in Iceland 1987–1991 [70]. Over the past decades a decline in child unintentional injury deaths rates has been reported within studies from Finland, mostly attributed to the decrease in the leading cause, i.e. fatal RTC [69,71]. The A&E rate in Belgium in 2005 was 11900/100,000 – data from Paediatric trauma registry [72]. In Romania the A&E rate for non-fatal injuries over 1999–2003 was estimated at 197/100,000 age <5 y, 140/100,000 age 5–14 y and 135/100,000 age 15–18 y – surveillance data from one hospital [73]. Using multiple data sources, the incidence rate of any injury for children in Velestino town, Greece was 28200/100,000 person year (6300/100,000 for injuries ISS>4) 1994/1995 [74]. The highest rates were in boys 10–14 y old, i.e. 38100 per 100,000 person year and children with younger parents/low income, i.e. OR = 1.33/1.37, p = 0.005/0.03 [74] and among migrant children [75]. All the studies underline boys and/or older children age 10–14 y as being at higher risk for both fatal and non-fatal injuries [69-77].

In the general population, all injury mortality rates vary from 23.8 per 100,000 (1998/1999, Italy [78]) to 126/100,000 (1990–2002, Lithuania [79]). Stable trends over 1990–2002 in mortality rates in Sweden have been shown in the literature reviewed whereas in Baltic communities, over the same study period, the rates registered a tendency to increase until 1994 followed by more stable patterns since 1997 [79]. Incidence rates for all injury treated ranged from 12900/100,000 in Greece 2001 (data from the surveillance system [80]) to 11400/100,000 in Norway 1985/1986 (data based on A&E attendances, hospitalization and general practitioners visits [81]) and 7470/100,000 in Spain 1990/1991 (A&E questionnaire data [82]). Incidence rate of severe injuries (ISS>15) is available from one study, i.e. 52,2 per 100,000 1998/1999 in Italy (data including pre-hospital deaths) [78]. Except in the over 65 age group, the injury incident rates in male exceeded those in females [81,83]. Falls and RTC were the prevailing mechanism for both fatal and non-fatal injuries (e.g. national data 1950–1995, The Netherlands [84], RTC 42% of injury deaths, Trondheim, Norway, 1985/1986 [81]; falls 40% and RTC 27% of all reported cases, Aquitaine, France 1985/1986 [83]; RTC over 75% of all injury ISS>16 in both Friuli Venezia, Giulia, Italy 1998/1999 and Groningen, The Netherlands, 1985–1990 [78,85] and 41.3% in Germany, 1997–2003 [86]). Falls are replacing RTC as a leading cause of injury deaths over 1971–2003, Finland [87]. Fractures, dislocations, contusions are among the most common causes of injury hospitalisations in Norway and Finland [81,88]. Injury Severity Scores show variation by external cause of injury (i.e. highest ISS values for accidents by firearms, RTC and burns [83]) and age group (i.e., the proportion of injures ISS>8 vary from 37.3% (children 0–17 y) to 8% (adults >14 y) [72,82]; over half of injuries ISS>15 are in the category 16–25 y [78]). Around 80% of all hospital admissions and general practitioners visits are injury AIS 1 [81].

## Discussion

The literature review has identified a significant body of epidemiological research in relation to injury mortality and morbidity.

### Data sources

Official statistics on injury mortality have been used in some research papers that offer national (e.g. England and Wales) representative data [e.g., [40,43,44,46]; however, these are only the tip of the iceberg on injury data or the first level of injury pyramid (i.e., deaths/hospitalisations/A&E attendances/general practitioners data/self reported). Hospital discharge data alone does not contain information on injury severity and has also the disadvantage of multiple counting for transfers from one unit to another or for readmissions for the same injury. Hospital as well as mortality data from the Central Statistics Office in Ireland have allowed description of injury epidemiology [67,68]. Although the authors managed to control for double counting of transfers, multiple admissions for the same injury were not reliably detected.

The issue of multiple counting due to several treatments for the same injury applies also to A&E departments' data. It is noteworthy in this context that in a study in West Glamorgan County, Wales, data from emergency departments are being used that are based on a 'diagnostic filter', so that only first attendances due to a new injury are included [57]. Use of trauma registry data in providing epidemiological pictures of injury occurrence is highly desirable, although some limitations of these data sources have been acknowledged. These include lack of information on death or injury before reaching the hospital and the selection criteria for entry into a trauma register. Trauma registers tend to include more severe trauma patients as determined by the length of stay of 2, 3 or 4 and more days [89]. On the other hand trauma registries, with the costs of data collection shared by participating hospitals, allow more appropriate codification of injury (i.e. Abbreviated Injury Scale), categorization by severity (i.e. ISS), exclude multiple admissions for the same injury, permit tracing of transfers, and capture information on post discharge deaths [e.g. [21]]. Moreover, there are trauma registries that include deaths on the scene, for example the French Rhone Road trauma registry covering injuries from road crashes in the Rhone county [90] or The PaEdiatric Network around TraumA registry that provides population-based data of injury in children and youngsters for the Flemish region [72]. North American literature shows that combining trauma registry data with pre-hospital deaths (coroner's department or medical examiner's data [91-93]) allows computation of major trauma death and morbidity rates. Some others authors use questionnaires to collect information on socio-demographic characteristics of the patient and injury details in studies of children under treatment or admitted to a hospital [76], patients admitted to a hospital and a sample of patients treated by general practitioners [81], or children treated at an emergency department only [58,75]. Questionnaires of self-reported illness as a primary data source have the advantage that the items to be collected can be tailored to specific research questions, but are time consuming to administer and usually involve smaller sample size compared to secondary data (data collected by people other than the researcher in question, for example, public vital statistics records). A further disadvantage is recall bias amongst respondents.

### The epidemiological profile

The identified studies from UK [40,42-46,48] and Europe [69,71] over the past decades indicate that child death rates from unintentional injury have decreased. The changes are mainly attributed to the decrease in RTC (trauma care improvements and traffic safety programmes [48,69,71] and changes in travel patterns (declines in walking and cycling activities – based on national data, England and Wales, 1985–1995 [43,44]). There are no significant trends in children for change in incidence of intentional deaths rates [42,48,69]. In this context two studies from North America are noteworthy that show a similar pattern of decreasing rates of child injury deaths based on RTC decline [94,95]. In USA homicide account for 36% of child injury deaths overall [94] whereas the same figure for UK is 3% [48]. Trends for child non-fatal injury rates are available from Scandinavian studies that shows little changes over time [70,71]. Child A&E rates in Belgium in 2005 [72], i.e. 11900/100,000 are lower compared with data reported in UK within previous time periods, i.e. 21490/100,000 and 13820/100,000 [50,53].

A comparison of child injury pyramids (deaths/hospitalisations/A&E attendances) in the UK [50] with data from Australia [96]/USA [97]/Canada [98] suggests the highest child death rates in USA, i.e. 1/150/1947 (UK); 1/77/524 (Australia, unintentional injuries only), 1/45/1300 (USA) and 1/73/1612 (Canada).

Overall in the general population there has been a decrease in fatal injury rates in studies from UK [65,66] and Europe [84,87] as a result of declining fatal RTC. The changes in fatal injuries have been attributed to trauma care improvements and safety measures [65,66] as well as to economic and 'autonomous' factors (i.e. demographic, socio-cultural and technological trends [84]). Socio-economic, historical and political factors have been used to explain the differences in mortality rates and trends between communities in Sweden and Baltic States [79]. Also in USA has been reported a decline in fatal injuries rates; however, it is noteworthy that intentional injuries here account for up to 42% of all injury deaths in USA, violence being a major cause [91]. According to the International Collaborative Effort on Injury Statistics data, firearm-related deaths among male 15–24 y in USA are up to 5/50 times higher when compared to Australia& New Zealand/England &Wales, 1992–1995 [99]. The mortality rate in UK 1988–1991, i.e., 35.0/100,000 [59] was found to be lower compared to data published in Lithuania, i.e. 126/100,000, Latvia 112/100,000 and Estonia 101/100,000 and much closer to data from Sweden, i.e., 38.0/100,000 1990–2002 [79].

Hospital admissions rates in the general population have increased in a study from Finland [88] with falls becoming the leading cause instead of RTC. Whereas no trends were available from studies in UK, a similar upward trend has been shown in a study from Australia [100]. These changes are attributed to preventive measures. The incidence of serious injury (ISS>15) varied from 19.2/100,000 (blunt injuries only, no pre-hospital deaths) in the UK [64] to 52.2/100,000 (pre-hospital death included) in Italy [78]. No major trauma population based rates are available within well defined populations across UK over recent time periods. The literature elsewhere shows serious injury rates of 71.5/100,000 (adult population, ISS>12 and pre-hospital death included) in Canada [93] and 25.6/100,000 (pre-hospital death excluded) in Australia [101]. It is hard to make direct international comparisons reliable because of inconsistencies in adjustment for age, inclusion of pre-hospital deaths and use of ISS to define injuries ISS>15 (Table 11). The injury pyramid in the general population in UK expressed either as (deaths/hospitalisations/any medical treatment) 1/45/630 [5] or (deaths/hospitalisation) 1/39 Ireland [67] and 1/30 England [59] by comparison with data available from USA 1/10/178 [102] and Australia 1/45/267 [103] (deaths/hospitalisations/A&E) again suggests the highest injury death rates in the USA. The pyramids also shows higher number of (hospitalisation or A&E) non-fatal injuries corresponding to one fatality in children [96-98] vs. general population [5,59,67,102,103] or adult population (i.e., 1/6/133 [82]).

**Table 11:** International comparisons of injury population – based rates of major trauma

**Author and** **date**	**Population size (million)**	**Place/time period**	**Adjust. for age**	**Inclusion of pre-hospital deaths**	**Description of the numerator**	**Rate** **(100,000 per year)**
Walsh et al. 1996 [50]	0.05*	Newcastle upon Tyne, UK/1990	N	Y	Injuries ISS>8	430
Petridou et al.2005 [74]	0.001**	Velestino, Greece/1994–1995	N	Y	Injuries ISS>4	6300
Gorman et al. 1995 [64]	3.2	Mersey Region and North Wales, UK/1989–1990	N	N	Blunt injuries ISS>15	19.0
McNicholl & Cooke 1995 [63]	1.5	Northern Ireland/1990–1991	N	N	Injuries ISS>15	23.2
Di Bartolomeo et al. 2004 [78]	1.2	Friuli Venezia Giulia, Italy/1998–1999	N	Y	Injuries ISS>15	52.2
Demetriades et al. 1998 [92]	9.4	Los Angeles, USA/1996	Y	Y	All trauma cases in the registry	151.0
Potenza et al. 2004 [91]	2.6	San Diego, USA/1987–1997	Y	Y	All trauma cases in the registry	195.0
Karmali et al. 2005 [93]	> 1***	Calgary, Canada/1999–2002	N	Y	Injuries ISS>12	71.5
Cameron et al. 1995 [101]	4.2	Victoria, Australia/1992–1993	N	N	Injuries ISS>15	25.6

*children <16 y; **children<14 y;*** adults ≥ 16 y

All the studies in which data have been analysed from a gender perspective alone show that both fatal and non-fatal injuries occurred more frequently in males than females. Whereas no consistent results for children age groups at higher risk, a well-recognised pattern of higher fatal and serious injury rates in males up to 65 y old then in females ≥ 65 years, has been shown in the papers reviewed [59,61,81,83]. Peak values of all injuries in males aged 15–24 y and 65+y and in females aged 65+y have been shown in the literature in UK [59] and elsewhere [83,102]. In the general population as well as in children RTC and falls are the predominant mechanisms for both fatal and non-fatal injuries. Moreover, higher RTC and assault rates in males compared to females have been reported [59,83,102]. Within a study in UK [61] sport and leisure activities were the third (male) and fourth (female) cause of injury after home, work/school and street/public locations whereas in Belgium 53.8% of A&E child injuries were sport related [72]. Disparities by race/ethnicity and (especially for fatal injuries) by SES persist over time. Fractures, dislocations and sprains, stains are cited as the most common causes of hospital admissions and A&E attendances [59,61,81,88].

### Injury pyramid

Many studies rely solely on mortality rates from injury for describing epidemiology [e.g. [42-44,46,69,87]]. Some investigators have used data on hospitalisations or A&E admissions only [e.g. [41,57,58,85]] but there are only a few studies giving data on deaths, hospitalisations and A&E attendance [50,64,84]. Ideally the epidemiological picture should include all levels of the injury pyramid. On the other hand, most minor injuries are difficult to estimate, unless through a specific survey, since most people with minor injuries will not present to health care services. To describe the total burden of injury within a particular community would require specific studies that are time-consuming and expensive to mount. As a minimum, we suggest that researchers should describe injury burden in terms of fatal and non-fatal serious injury.

Key issues in computing incidence rates are representativeness and generalisability. There are only a few studies that have used large national databases to estimate national injury rates (e.g. Patients Episodes Database for Wales [60]; Road Traffic Accident Registry, Occupational Registry, National Medical Registry and Dutch Central Bureau of Statistics in The Netherlands [84]; National Hospital Discharge Register in Finland [69,71,87,88]). Many of the articles we reviewed focused on particular communities usually defined by geographic areas, with analysis extended from one to several years. Some were based on proportions (socio-demographic characteristics and injury details) rather than rates [e.g. [77,85]]. When seeking to extrapolate results from certain population sub-groups to the general population researchers consider carefully the limitations e.g. the socio-demographic structure of the population studied compared with the general population. To discern time trends analyses should include more than two year's worth of data where available. Finally proportions should complement rates in any descriptive epidemiological paper dealing with injury occurrence, rather than be a substitute for them.

### Matching numerator and denominator

The definition of appropriate numerators and denominators is crucial for calculating population-based rates. This issue applies not only to injury research but also to the larger area of population based epidemiology. A number of approaches to determine the denominator or 'population at risk' have been described within the literature presenting data from the general practice [104,105] or from sentinel practice networks [106,107]. Whereas use of the number of consultations (i.e., yearly or weekly contact group) has proved to be the step forward to determine the population practice, the ideal denominator seems to be the total population within a clearly defined geographic area, i.e., the practice catchment area [107]. Within this review, most of the articles simply use as the numerator the number of discharges and/or A&E visits from hospitals located in a study area. The denominator is the regional census population. For example one study provides rates for the town of Trondheim based on hospital discharges from one hospital that is judged to be the only provider for the region [81]. However, a number of researchers acknowledge that population movement occurs resulting in a possible mismatch between numerator and denominator. For example in one study from UK [61] the authors tried to account for the admissions of area residents outside their area (i.e. postcode EH45 – Livingstone town), although the exact figures were unavailable. In a remote area in France [83] researchers took into account area cases diagnosed outside region by extrapolation based on mortality rates. The authors themselves recognised the possibility of an inaccurate estimate. However, in this study they attempted to include commuter populations in their rate estimates. Some other studies exclude out-of-area residents treated at area hospitals as well as out-of-area hospitalisation of area residents when defining numerator [78]. The ideal solution to these methodological inconsistencies would be a small area analysis in the form of aggregation based on home address of the patients and of the census population in a corresponding way (e.g. aggregation by electoral wards over Wales [60]). However, this is not a viable option when the database is restricted to information from a limited number of hospitals. This is the case of data provided by national registries [e.g., [21]] that relies on a selection of hospital reporting on a voluntary basis; regional registries using patient inclusion criteria based on place of residence [93] or place of injury occurrence [78,90,91] or hospital discharge databases. We suggest that, when possible, researchers should consider all cases from area residents in constructing a numerator. Further research should focus on development of an appropriate methodological approach to deal with this issue.

Another issue in the context of defining numerators and denominators for further calculation of population based rates is related to data quality. Although completeness and accuracy of the records fields within trauma registries have rarely been reported in the literature, there is evidence that validated trauma registries are a reliable data source for population based epidemiology [108,109]. Apart from the validity, an other component of registry data quality is the completeness of case ascertainment defined as the proportion of all cases in the target population that appear in the data base [110]. Case ascertainment rates of 90% of all major trauma (injuries ISS>15) for Victoria State trauma registry [111] or 87% of serious injuries (NISS9+) for French Rhone road trauma registry [90] have been shown in the literature. Use of methods such as capture-recapture through record linkage between several data sources (for example, within road injury research, police records-trauma registry [112], police records-hospitalisation records [113] and hospitalisation records-police records, deaths certificates, trauma registry [114]) can overcome incomplete registration of injuries and provide extensive scientific data for population based epidemiologic research. The capture-recapture method ca be applied when several data sources are available and under certain circumstances (i.e., for the traditional two sample method – independent samples, closed population, homogeneity of capture and perfect matching) [115,116]. It has been used in injury research [e.g., [112-114,117]] as well as within larger area of epidemiology [116]. Within injury research, the variety of data sources (e.g., death certificates, hospitalisation discharges, trauma registry data, ambulance records, physician's records, survey data, police records) can only be in favour of the widespread application of this technique in assessing data quality and estimating injury population-based rates.

### Measures of injury severity

We reviewed an extensive number of articles especially from UK; however, only few of them presented data using a specific measure of injury severity. Some papers refer to categorisation of injuries by severity based on non-specific general measures: mortality, hospitalisations and A&E admissions [e.g., [80]]. However, death is an injury outcome rather than a severity measure, whereas hospitalisations and A&E attendances are subjective measures (for a detailed discussion related to non-specific measures of injury severity see the review by Beattie and al [29]). Some other papers used different measures. For example in one study severity is arbitrarily defined by four categories based on nature of injury and treatment [53] whereas in another study 'sever trauma' is defined by length of stay over 48 hours including all non-survivors [72]. Other authors used case-fatality rates as a function of injury severity [84] or fractures as an indicator of severity [57]. This is probably explained by data limitations – the researchers analysing routinely collected data or primary data lacking information on injury severity. Injury Severity Score remains the most widely accepted measure of severity in the field of injury, although some limitations of this measure have previously been acknowledged. Without a standard severity coding it is difficult to define clearly the category of major trauma (i.e. ISS ≥ 16) for those requiring more attention in terms of both prevention and care, to interpret injuries and to make comparisons across studies. Walsh and Jarvis [55] have pointed out that any epidemiological study using ISS to select patients with more severe injury may overcome issues related to 'selection bias' (i.e. factors apart from the injury itself influencing health care use such are hospital policies, bed supply, SES of the patient) that applies both to hospital admissions and A&E attendances.

### Relationship of injury to socio-economic status (SES)

Only one study outside UK has analysed the relationship of rates of injury to SES [74]. Whereas a straightforward inverse association between injury death rates and SES has been observed from the literature review, the evidence for socioeconomic inequalities and injury morbidity has not been wholly consistent. For fatal injuries socioeconomic gradients or inverse association with SES [40,42,46,48] have been shown in descriptive studies of childhood injuries. For non-fatal injuries results vary from presenting no relationship [57,62] to associations [50,55,58,61,74] of injury requiring hospitalisation and/or A&E visits with SES. Evidence comes mostly from ecological studies (children alone as well as from the general population) that are limited by the 'ecological fallacy'. It is noteworthy researchers have been used complex measures of socio-economic status (e.g. Townsend deprivation score, Jarman 8 Index, Carstairs Depcat Score) or one indicator only (e.g. parent education, insurance status, social class). Either measured at an individual level or area based, SES remains a multidimensional concept, subject to confounding variables such as race/ethnicity (a detailed, complex description on the issue of injury – SES can be found in the literature review by Cubbin & Smith [38]). Finally, we would recommend the researchers to use adequate, complex measures for SES and when possible to apply an analytical design to confirm their findings.

## Conclusion

In this paper we reviewed articles dealing with all injury epidemiology expressed as mortality and/or morbidity rates. Some methodological considerations have been discussed as well as possible solutions to the difficulties in counting injuries and constructing population-based rates. New methodological approaches should be developed to deal with the study design inconsistencies and the knowledge gaps identified across this review. Probably, in future, population-based trauma registries collecting data from all trauma hospitals within a larger region will be used and will allow information on severity as defined by the ISS.

## Abbreviations

**A&E**: (Accident & Emergency); **AIS**: (Abbreviated Injury Scale); **ISS**: (Injury Severity Score); **HES**: (Hospital Episodes Statistics); **RTC**: (Road Traffic Crashes); **SES**: (socioeconomic status); **TARN**: (Trauma Audit and Research Network).

## Competing interests

The authors declare that they have no competing interests.

## Authors' contributions

RA and FL designed the study; RA performed the literature review and drafted the manuscript; FEL contributed to the written manuscript and supervised the study. SJO provided feedback and contributed to the written manuscript. All authors read and approved the final manuscript.

## Pre-publication history

The pre-publication history for this paper can be accessed here:



## References

[B1] Krug EG, Sharma GK, Lozano R (2000). The Global Burden of Injuries. Am J Public Health.

[B2] Murray CL, Lopez AD (1997). Alternative projections of mortality and disability by cause 1990–2020. Lancet.

[B3] Van Beeck EF, Van Roijen L, Mackenbach JP (1997). Medical Costs and Economic Production Losses due to Injuries in the Netherlands. J Trauma.

[B4] Bastida JL, Aguilar PS, González BD (2004). The Economic Costs of Traffic Accidents in Spain. J Trauma.

[B5] British Medical Association (2001). Injury prevention.

[B6] Secretary of State for Health (1998). Our Healthier Nation: A contract for health.

[B7] Secretary of State for Health (1999). Saving Lives: Our Healthier Nation.

[B8] Information and Statistics Division Scotland National Statistics, Department of Trade and Industry Fireworks Injuries Enquiry.

[B9] Office for National Statistics (2002). Health Survey for England 2000.

[B10] Office for National Statistics (2003). Health Survey for England 2001.

[B11] McCormick A, Fleming D, Charlton J (1995). Morbidity statistics from general practice: fourth national study 1991–1992 Series MB5 No 3 OPCS.

[B12] Department of Trade and Industry (2003). 24th (final) Report of the Home and Leisure Accident Surveillance System 2000, 2001, 2002 data.

[B13] European Injury Database. https://webgate.ec.europa.eu/idbpa/welcome.jsp.

[B14] Hospital Episodes Statistics 2006/2007. http://www.dh.gov.uk/en/Publicationsandstatistics/Statistics/HospitalEpisodeStatistics/DH_576.

[B15] Jones S, Parry S Burden of disease – injuries on overview.

[B16] Office for National Statistics (2007). Mortality Statistics: Injury and Poisoning 2005 Series DH4 No 30.

[B17] Office for National Statistics (2001). Geographic Variations in Health – Decennial Supplement DS 16.

[B18] Christensen MC, Ridley S, Lecky FE, Munro V, Morris S (2008). Outcomes and costs of blunt trauma in England and Wales. Crit Care.

[B19] Cryer C, Langley JD, Stephenson SCR, Jarvis SN, Edwards P (2002). Measure for measure: the quest for valid indicators of non-fatal injury incidence. Public Health.

[B20] McClure RJ, Peel N, Neale R (2002). Appropriate indicators for injury control?. Public Health.

[B21] Trauma Audit and Research Network. https://www.tarn.ac.uk/Login.aspx.

[B22] Committee on Injury Scaling, Association for the Advancement of Automotive Medicine (1990). The Abbreviated Injury Scale 1990 Revision.

[B23] Committee on Injury Scaling, Association for the Advancement of Automotive Medicine (1985). The Abbreviated Injury Scale.

[B24] Committee on Injury Scaling, Association for the Advancement of Automotive Medicine (1998). The Abbreviated Injury Scale 1990 Revision Update 98.

[B25] Osler T, Baker SP, Long W (1997). A modification of the injury severity score that both improves accuracy and simplifies scoring. J Trauma.

[B26] Osler T, Rutledge R, Deis J, Bedrick E (1996). An international classification of disease-9 based injury severity score. J Trauma.

[B27] Wong SNS, Leung KK (2008). Injury Severity Score (ISS) vs. ICD-derived Injury Severity Score (ICISS) in a patient population treated in a designated Hong Kong trauma centre. MJM.

[B28] Tepas JJ, Molit DC, Talbert JL (1987). The pediatric trauma score as a predictor of injury in the injured child. J Pediatr Surg.

[B29] Beattie TF, Currie CE, Williams JM, Wright P (1998). Measures of injury severity in childhood: a critical overview. Inj Prev.

[B30] Champion HR, Sacco WJ, Lepper RL, Atzinger EM, Copes WS, Prall RH (1980). An anatomic index of injury severity. J Trauma.

[B31] Seow-Yian T, Sloan EP, Zun L, Zaret P (2004). Comparison of the New Injury Severity Score and the Injury Severity Score. J Trauma.

[B32] Linn S (1995). The Injury Severity Score: importance and uses. Ann Epidemiol.

[B33] Moore L, Clark DE (2008). The value of trauma registries. Injury.

[B34] Stephenson S, Henley G, Harrison JE, Langley JD (2004). Diagnosis based injury severity scaling: investigation of a method using Australian and New Zealand hospitalisations. Inj Prev.

[B35] Kim Y, Jung Y, Kim C (2000). Validation of the international classification of diseases 10^th ^edition – based injury severity scores (ICISS). J Trauma.

[B36] Cryer C (2006). Severity of injury measures and descriptive epidemiology. Inj Prev.

[B37] Baker SP, O'Neill B, Haddon W, Long WB (1974). The Injury Severity Score: a method for describing patients with multiple injuries and evaluating emergency care. J Trauma.

[B38] Cubbin C, Smith GS (2002). Socioeconomic Inequalities in Injury: Critical Issues in Design and Analysis. Annu Rev Public Health.

[B39] Cummings P, Koepsell TD, Mueller BA (1995). Methodological Challenges in Injury Epidemiology and Injury Prevention Research. Annu Rev Public Health.

[B40] Avery JG, Vaudin JN, Fletcher JL, Watson JM (1990). Geographical and Social Variations in Mortality Due to Childhood Accidents in England and Wales 1975–1984. Publ Health.

[B41] Edwards SP, Green J, Roberts I, Lutchmun S (2006). Deaths from injury in children and employment status in family: analysis of trends in class specific deaths rates. BMJ.

[B42] Roberts I, Li L, Barker M (1998). Trends in intentional injury deaths in children and teenagers (1980–1995). J Public Health Med.

[B43] DiGuiseppi C, Roberts I, Li L (1997). Influence of changing travel patterns on child death rates from injury: trend analysis. BMJ.

[B44] DiGuiseppi C, Li L, Roberts I (1998). Influence of travel patterns on mortality from injury among teenagers in England and Wales, 1985–95: trend analysis. BMJ.

[B45] Roberts I, Campbell F, Hollis S, Yates DW (1996). Reducing accident death rates in children and young adults: the contribution of hospital care. BMJ.

[B46] Roberts I, Power C (1996). Does the decline in child injury mortality vary by social class? A comparison of class specific mortality in 1981 and 1991. BMJ.

[B47] Armstrong AM, Robson WJ (1992). Deaths from Injury and Poisoning of Children in Liverpool: A Ten-Year Survey 1978–1987. Publ Health.

[B48] Roberts I, DiGuiseppi C, Ward H (1998). Childhood injuries: extent of the problem, epidemiological trends, and costs. Inj Prev.

[B49] Office of population censuses and Surveys (1980). Classification of Occupations.

[B50] Walsh SS, Jarvis SN, Towner EM, Aynsley-Green A (1996). Annual incidence of unintentional injury among 54 000 children. Inj Prev.

[B51] Edwards SP, Green J, Lachowycz K, Grundy C, Roberts I (2008). Serious injuries in children: variation by area deprivation and settlement type. Arch Dis Child.

[B52] Graham CA, MacDonald A, Stevenson J (2005). Children's injuries in a Scottish district general hospital. Injury.

[B53] Laing GJ, Logan S (1999). Patterns of unintentional injury in childhood and their relation to socio-economic factors. Publ Health.

[B54] MacInnes K, Stone DH (2008). Stages of development and injury: An epidemiological survey of young children presenting to an emergency department. BMC Public Health.

[B55] Walsh SS, Jarvis SN (1992). Measuring the frequency of 'severe' accidental injury in childhood. J Epidemiol Community Health.

[B56] Hippisley-Cox J, Groom L, Kendrick D, Coupland C, Webber E, Savelyich B (2002). Cross sectional survey of socioeconomic variations in severity and mechanism of childhood injuries in Trent 1992–7. BMJ.

[B57] Lyons RA, Lo SV, Heaven M, Littlepage BN (1995). Injury surveillance in children – usefulness of a centralised database of accident and emergency attendances. Inj Prev.

[B58] Ness V, Hoskins R, Robb A (2002). The use of childhood injury surveillance within a general accident and emergency department. Accid Emerg Nurs.

[B59] Cryer PC, Davidson L, Styles CP, Langley JD (1996). Descriptive epidemiology of injury in the South East: identifying priorities for action. Publ Health.

[B60] Lyons RA, Jones SJ, Deacon T, Heaven M (2003). Socioeconomic variation in injury in children and older people: a population based study. Inj Prev.

[B61] Gorman DR, Ramsay LJ, Wilson GS, Freeland P (1999). Using routine accident and emergency data to describe local injury epidemiology. Publ Health.

[B62] McKee CM, Gleadhill DN, Watson JD (1990). Accident and emergency attendance rates: variation among patients from different general practices. Br J Gen Pract.

[B63] McNicholl B, Cooke RS (1995). The epidemiology of major trauma in Northern Ireland. Ulster Med J.

[B64] Gorman DF, Teanby DN, Sinha MP, Wotherspoon J, Boot DA, Molokhia A (1995). The epidemiology of major injuries in Mersey Region and North Wales. Injury.

[B65] Lecky FE, Woodford M, Bouamra O, Yates DW (2000). Trends in trauma care in England and Wales 1989–97. UK Trauma Audit and Research Network. Lancet.

[B66] Lecky FE, Woodford M, Bouamra O, Yates DW (2002). Lack of change in trauma care in England and Wales since 1994. Emerg Med J.

[B67] Scallan E, Staines A, Fitzpatrick P, Laffoy M, Kelly A (2004). Unintentional injury in Ireland: a comparison of mortality and morbidity data. J Public Health (Oxf).

[B68] Boland M, Staines A, Fitzpatrick P, Scallan E (2005). Urban-rural variation in mortality and hospital admission rates for unintentional injury in Ireland. Inj Prev.

[B69] Mattila VM, Parkkari J, Niemi S, Kannus P (2005). Injury-related deaths among Finnish adolescents in 1971–2002. Injury.

[B70] Stefansdottir A, Mogensen B (1997). Epidemiology of childhood injuries in Reykjavik 1974–1991. Scand J Prim Health Care.

[B71] Parkkari J, Kannus P, Niemi S, Koskinen S, Palvanen M, Vuori I (2000). Childhood deaths and injuries in Finland in 1971–1995. Int J Epidemiol.

[B72] Voorde P Van de, Sabbe M, Calle P, Lesaffre E, Rizopoulos D, Tsonaka R, Christiaens D, Vantomme A, De Jaeger A, Matthys D (2008). Paediatric trauma and trauma care in Flanders (Belgium). Methodology and first descriptive results of the PENTA registry. Eur J Pediatr.

[B73] Oprescu F, Peek-Asa C, Young T, Figan I, Nour D (2008). Emergency department visits for non-fatal childhood injuries in Romania. Eur J Emerg Med.

[B74] Petridou E, Anastasiou A, Katsiardanis K, Dessypris N, Spyridopoulos T, Trichopoulos D (2005). A prospective population based study of childhood injuries: the Velestino town study. Eur J Public Health.

[B75] Petridou E, Moustaki M, Gemanaki E, Djeddah C, Trichopoulos D (2001). Intentional childhood injuries in Greece 1996–97 – Data from a population-based Emergency Department Injury Surveillance System (EDISS). Scand J Public Health.

[B76] Brudvik C (2000). Child injuries in Bergen Norway. Injury.

[B77] Borzecki A, Lukawski K, Robak L, Borzecka H, Sieklucka-Dziuba M (2002). Causes and circumstances of injuries in children. Ann Univ Mariae Curie Sklodowska Med.

[B78] Di Bartolomeo S, Sanson G, Michelutto V, Nardi G, Burba I, Francescutti C, Lattuada L, Scian F, The Regional Study-Group on Major Injury (2004). Epidemiology of major injury in the population of Friuli Venezia Giulia – Italy. Injury.

[B79] Ekman R, Kaasik T, Starkuviene S, Bangdiwala SI (2007). Injury mortality in local communities in Sweden and in three Baltic States: implications for prevention. Int J Inj Ctrl Safety Promotion.

[B80] Petridou E, Dessypris N, Frangakis CE, Belechri M, Mavrou A, Trichopoulos D (2004). Estimating the population burden of injuries: a comparison of household surveys and emergency department surveillance. Epidemiology.

[B81] Sahlin Y, Stene TM, Lereim I, Balstad P (1990). Occurrence of injuries in a defined population. Injury.

[B82] Plasencia A, Borelli C (1996). Population-based study of emergency department admissions and deaths from injuries in Barcelona, Spain: Incidence, causes and severity. Eur J Epidemiol.

[B83] Tiret L, Garros B, Maurette P, Nicaud V, Thicoipe M, Hatton F, Erny P (1989). Incidence, causes and severity of injuries in Aquitaine, France: a community – based study of hospital admissions and deaths. Am J Public Health.

[B84] Van Beeck EF, Looman CWN, Mackenbach JP (1998). Mortality due to unintentional injuries in The Netherlands, 1950–1995. Public Health Rep.

[B85] Sluis CK Van der, Klasen HJ, Eisma WH, Ten Duis HJ (1996). Major Trauma in Young and Old: What is the Difference?. J Trauma.

[B86] Buschmann C, Kuhne CA, Losch C, Kolb DN, Ruchholtz S (2008). Major trauma With Multiple Injuries in German Children. A Retrospective review. J Pediatr Orthop.

[B87] Kannus P, Niemi S, Palvanen M, Parkkari J, Järvinen M (2005). Secular trends in rates of unintentional injury deaths among adult Finns. Injury.

[B88] Kannus P, Niemi S, Parkkari J, Palvanen M (2001). Epidemiology of adulthood injuries: A quickly changing profile in Finland. J Clin Epidemiol.

[B89] Horan JM, Mallonee (2003). Injury Surveillance. Epidemiol Rev.

[B90] Laumon B, Martin JL, Collet P, Chiron M, Verney MP, Ndiaye A, Vergnes I (1997). A French road trauma registry: first results.

[B91] Potenza BM, Hoyt DB, Coimbra R, Fortlage D, Holbrook T, Hollingswoth-Fridlung P, the Trauma Research and Education Foundation (2004). The epidemiology of serious and fatal injury in San Diego County over an 11-year period. J Trauma.

[B92] Demetriades D, Murray J, Sinz B, Myles D, Chan L, Sathyaragiswaran L, Noguchi T, Bongard FS, Cryer GH, Gaspard DJ (1998). Epidemiology of Major Trauma and Trauma Deaths in Los Angeles County. J Am Coll Surg.

[B93] Karmali S, Laupland K, Harrop AR, Findlay C, Kirkpatrick AW, Winston B, Kortbeek J, Crowshoe L, Hameed M (2005). Epidemiology of severe trauma among status Aboriginal Canadians: a population-based study. CMAJ.

[B94] Bernard SJ, Paulozzi LJ, Wallace LJD (2007). Fatal injuries among children by race and ethnicity–United States, 1999–2002. MMWR Surveill Summ.

[B95] Pan SY, Desmeules M, Morrison H, Semenciw R, Ugnat AM, Thompson W, Mao Y (2007). Adolescent Injury Deaths and Hospitalisation in Canada: Magnitude and Temporal Trends (1979–2003). J Adolesc Health.

[B96] Nolan T, Penny M (1992). Epidemiology of non-intentional injuries in an Australian urban region: results from injury surveillance. J Paediatr Child Health.

[B97] Gallagher SS, Finison K, Guyer B, Goodenough S (1984). The incidence of injuries among 87,000 Massachusetts children and adolescents: results of the 1980–1981 Statewide Childhood Injury Prevention Program Surveillance System. Am J Public Health.

[B98] Spady DW, Saunders DL, Schopflocher DP, Svenson LW (2004). Patterns of injury in children: a population based approach. Pediatrics.

[B99] MacKenzie EJ (2000). Epidemiology of Injuries: Current Trends and Future Challenges. Epidemiol Rev.

[B100] Watt GM, Ozanne-Smith J (1996). Trends in public hospital injury admission rates, Victoria, July 1987 to June 1993. Aust N Z J Public Health.

[B101] Cameron P, Dziukas L, Hadj A, Clark P, Hooper S (1995). Major trauma in Australia: a regional analysis. J Trauma.

[B102] Vyrostek SB, Annest JL, Ryan GW (2004). Surveillance for Fatal and Nonfatal Injuries – United States, 2001. MMWR Surveill Summ.

[B103] Watson WL, Ozanne-Smith J (2000). Injury surveillance in Victoria, Australia: developing comprehensive injury incidence estimates. Accid Anal Prev.

[B104] Bartholomeeusen S, Kim CY, Mertens R, Faes C, Buntinx F (2005). The denominator in general practice, a new approach from the Intego database. Fam Pract.

[B105] Jenkins C, Campbell J (1996). Catchment areas in general practice and their relation to size and quality of practice and deprivation: a descriptive study in one London borough. BMJ.

[B106] Kellerhof M, Gritz K, Brand H (1995). Denominator estimation: approaches in the Hamburg paediatric sentinel network. J Epidemiol Community Health.

[B107] Schlaud M, Brenner MH, Hoopmann M, Schwartz FW (1998). Approaches to the denominator in practice based epidemiology: a critical overview. J Epidemiol Community Health.

[B108] Rodenberg H (1996). The Florida trauma system: assessment of a statewide database. Injury.

[B109] Datta I, Findlay C, Kortbeek JB, Hameed M (2007). Evaluation of a regional trauma registry. Can J Surg.

[B110] Goldberg J, Gelfand HM, Levy PS (1980). Registry evaluation methods: a review and case study. Epidemiol Rev.

[B111] Cameron PA, Gabbe BJ, McNeill JJ (2005). The trauma registry as a statewide quality improvement tool. J Trauma.

[B112] Amoros E, Martin JL, Laumon B (2007). Estimating non-fatal road causalities in a large French county, using the capture-recapture method. Accid Anal Prev.

[B113] Meuleners LB, Lee AH, Cercarelli R, Legge M (2006). Estimating crashes involving heavy vehicles in Western Australia, 1999–2000: A capture -recapture method. Accid Anal Prev.

[B114] Clark DE, Hahn DR (1999). Hospital Trauma Registries Linked with Population-Based Data. J Trauma.

[B115] International Working Group for Disease Monitoring and Forecasting (1995). Capture-recapture and multiple-record systems estimation I: History and theoretical development. Am J Epidemiol.

[B116] International Working Group for Disease Monitoring and Forecasting (1995). Capture-recapture and multiple-record systems estimation II: Applications in human diseases. Am J Epidemiol.

[B117] LaPorte RE, Dearwater S, Chang YF, Songer TJ, Aaron DJ, Anderson RL, Olsen T (1995). Efficiency and accuracy of disease monitoring systems: application of capture-recapture methods to injury monitoring. Am J Epidemiol.

